# Nuclear shift of hnRNP K protein in neoplasms and other states of enhanced cell proliferation

**DOI:** 10.1038/sj.bjc.6601250

**Published:** 2003-10-14

**Authors:** J Ostrowski, K Bomsztyk

**Affiliations:** 1 Department of Gastroenterology, Medical Center for Postgraduate Education, Maria Sklodowska-Curie Memorial Cancer Center, ul. Roentgena 5, 02-781 Warsaw, Poland; 2 Institute of Oncology, 02-781 Warsaw, Poland; 3 Department of Medicine, University of Washington, Seattle, WA 98195, USA

**Keywords:** hnRNP K protein, proliferation, hepatocyte culture, hepatectomy, hepatic neoplasms

## Abstract

The heterogeneous nuclear ribonucleoprotein K (hnRNP K), is a ubiquitously expressed protein that interacts with signal transducers, proteins that modulate gene expression and selective RNA and DNA motifs. K protein is modified in response to extracellular signals and directly regulates rates of transcription and translation. We used serum-treated hepatocyte culture, liver after partial hepatectomy and hepatic neoplasms as systems to compare expression, subcellular distribution and tyrosine phosphorylation of K protein in quiescent and dividing cells. The results show that expression of K protein mRNA was increased in states of enhanced proliferation. Levels of nuclear K protein were also higher in proliferating compared to resting cells. In contrast, levels of cytoplasmic K protein were the same or lower in dividing compared to quiescent cells. States of enhanced proliferation were also associated with increased levels of K protein tyrosine phosphorylation. Nuclear shift of K protein in dividing cells may reflect involvement of K protein in signalling multiple events that regulate expression of genes in proliferating cells.

The heterogeneous nuclear ribonucleoprotein K (hnRNP K) protein is an abundant and ubiquitous factor that interacts with a diverse types of molecules (Bomsztyk *et al*, 1997) including RNA (Dejgaard *et al*, 1994), DNA (Ostrowski *et al*, 1994), factors involved in chromatin remodelling (Denisenko and Bomsztyk, 1997; Shnyreva *et al*, 2000), transcription (Du *et al*, 1993), RNA splicing (Shnyreva *et al*, 2000) and translation (Bomsztyk *et al*, 1997). K protein also interacts with factors involved in signal transduction, including inducible kinases (Weng *et al*, 1994; Bustelo *et al*, 1995; Van Seuningen *et al*, 1995). The diversity of its interactions can be accounted for by K protein's modular structure (Bomsztyk *et al*, 1997). K protein is modified in response to changes in extracellular environment including cytokines, growth factors and oxidative stress (Ostrowski *et al*, 1991; 2000a). Changes in K protein phosphorylation regulate its interaction with nucleic acids and protein partners (Schullery *et al*, 1999; Ostrowski *et al*, 2000a; Ostareck-Lederer *et al*, 2002). These properties suggest that K protein bridges signal transduction pathways to nucleic acid-directed processes. In further support of this model, several studies have shown that K protein can alter rates of transcription (Michelotti *et al*, 1996) and translation (Ostareck *et al*, 1997).

K protein contains three K homology (KH) domains (Siomi *et al*, 1993) that are highly conserved in organisms as diverse as yeast and mammals. In *Drosophila*, the KH domain-containing product, *bancal*, is most closely related to the mammalian K protein. Null and weak alleles of *bancal* impair adult appendages morphogenesis (Charroux *et al*, 1999; Gates and Thummel, 2000). This phenotype reflects decreased cell proliferation in the imaginal disc cells (Charroux *et al*, 1999). Expression of human K protein rescues these fly phenotypes, suggesting that like the structure, K protein function has also been evolutionarily conserved.

Since *bancal* has been shown to affect cell proliferation in *Drosophila* (Charroux *et al*, 1999; Gates and Thummel, 2000), we wondered if K protein expression is altered in proliferating mammalian cells. To explore the role of K protein in mitogenic responses, we chose hepatic cell lineage because cell cultures and useful models of enhanced proliferative states in intact organs and cancer are available. Our study showed increased expression of K protein mRNA and a nuclear shift of K protein in cultured serum-treated hepatocytes, in regenerating livers postpartial hepatectomy and in hepatic neoplasms.

## MATERIAL AND METHODS

### Cells

Rat hepatoma cells expressing human insulin receptors, HTC-IR, were grown in plastic cell culture flasks in DME media supplemented with 10% FBS, 2 mM glutamine, penicillin (100 U ml^−1^), streptomycin (0. 01%), and humidified with 7/93% CO_2_/air gas mixture.

### Animals

Mice were housed under constant room temperature with a 12 : 12-h light–dark and permitted free access to water and to standard food pellets. The animals received humane care in compliance with the regulation of Cancer Center.

Experiments utilised CBA-T6/W mice, which had developed spontaneous hepatocellular neoplasms, and BALB/c mice with liver implanted L1 sarcoma tumors (Ostrowski *et al*, 2000c). L1 sarcoma cell line was propagated under standard conditions in MEM supplemented with 10% FCS and antibiotics. BALB/c mice, 2 months old, were anaesthetised with ether for laparotomy, and 10^5^ viable L1 cells suspended in 0.05 ml of PBS were injected under the capsule of left lateral lobe of the liver. After 3 weeks, animals with implanted hepatic tumours were used in these studies. Mice anaesthetised by ether were killed, the livers were rapidly resected and classified by gross examination into tumour and hepatic tissue. Two portions of each specimen were frozen in liquid nitrogen and stored in −80°C until use. The remaining portions were fixed in formalin and embedded in paraffin for histological examination.

Partial hepatectomy was performed under ether anaesthesia on 2-month-old BALB/c male mice. Under aseptic conditions animals were subjected to mid-ventral laparatomy and resection of the left lateral lobe which constitutes about one-third of the total liver. The removed lobe was immediately frozen in liquid nitrogen and stored at −80°C until use. After the indicated times, animals were killed and the remnant liver was rapidly collected and frozen. Sham-operated animals underwent mid-ventral laparatomy without resection of liver lobe.

### Northern blot analysis and RT–PCR

Total RNA was prepared from tissues and cell pellets using TRIzol reagent. A measure of 10 *μ*g of total RNA was denatured with formaldehyde–formamide and electrophoresed in a 1% agarose/formaldehyde gel. RNA was then transferred to Hybond N nylon membranes in 10 × SSC. The membranes were incubated at 65°C for 20 h with 2 × 10^7^ d.p.m. of the ^32^P-labelled DNA probes in the hybridisation buffer (6 × SSC, 0.5% Ficoll, 0.5% PVP, 0.5% BSA, 0.5% SDS, 100 *μ*g ml^−1^ herring sperm DNA). Excess probe was removed from the membrane by serial washes at 65°C in 1 × SSC/0.1% SDS. The hybridised probes were visualised by autoradiography.

A measure of 5 *μ*g of total RNA was reverse transcribed using Superscript II RT (GIBCO-BRL) and oligo-dT in 20 *μ*l volume as per the manufacturer's protocol. RT reactions were diluted 1 : 10 with water, and PCR was carried out as described previously (Ostrowski *et al*, 2003b) using 2 *μ*l of cDNAs and primers for *c-myc*. [*α*-^32^P]dCTP (NEN) was used to label the PCR products. PCR products were resolved on native 5% polyacrylamide gels, then the gels were dried and the PCR products were quantified using a phosphorimager. Densitometric analysis was performed using OptiQuant™ Image Analysis Software (Packard). The levels of band intensities after background subtraction were expressed in digital light units (DLU).

### Cell extracts

Cytoplasmic and nuclear extracts were prepared as described previously (Ostrowski *et al*, 1991,2000b). Separation of the nuclear fraction from the cytosol was monitored with the cytosolic enzyme marker lactic dehydrogenase and the purity of nuclear extracts was greater than 92%. The protein concentration was measured using MicroBCA protein assay, Pierce Biotechnology (Rockford, IL, USA).

### Electrophoresis and immunoblotting

Equal amounts of sample protein (50 *μ*g) were separated by 10% SDS–PAGE and immunostained by standard methods as described previously (Ostrowski *et al*, 1991,2000b).

### Cell proliferation

DNA synthesis was determined by the incorporation of ^3^H-thymidine. Exponentially growing HTC-IR cells were harvested, seeded at a density of 5 × 10^3^ per well in 96-well plates and then grown in DMEM containing 10% FBS. After 24 h, cells were made quiescent by 48 h serum deprivation and then they were treated with 15% FBS. Cell growth was monitored at 3, 6 and 24 by adding 0.5 *μ*Ci of ^3^H-thymidine to each well for 3 h. Finally, cells were harvested, DNA was collected on GFC filters, and the radioactivity was determined by scintillation counting. Three independent experiments were performed and all assays were repeated in octuplicate.

Mice with spontaneous hepatocellular neoplasms and those after sham or partial hepatectomy were given 25 *μ*Ci of ^3^H-thymidine intraperitoneally 1 h prior to euthanasia. Hepatic DNA was extracted by proteinase K digestion followed by phenol–chloroform extraction, and the quantity of the DNA was measured spectrometrically. The radioactivity of 100-*μ*g samples of total hepatic DNA was determined by scintillation counting.

### Quantification

Quantification analysis was performed either by photographing with a digital camera (DC40, Kodak) or scanning the intensity of the bands with an ImageScanner, Amersham Pharmacia Biotech or by scanning ^32^P radioactivity with a Phosphorimager apparatus. Densitometric analysis was performed using OptiQuant™ Image Analysis Software (Packard).

### Statistical analysis

Results are presented as means+s.d. Significant difference between mean values was assessed by means of analysis of variance (ANOVA). *P*-values for differences from control results were calculated using the Bonferroni method. Means were considered to be different if *P*<0.05.

## RESULTS

### Expression and subcellular localisation of K protein from serum-treated hepatocyte culture

**Figure 1:**
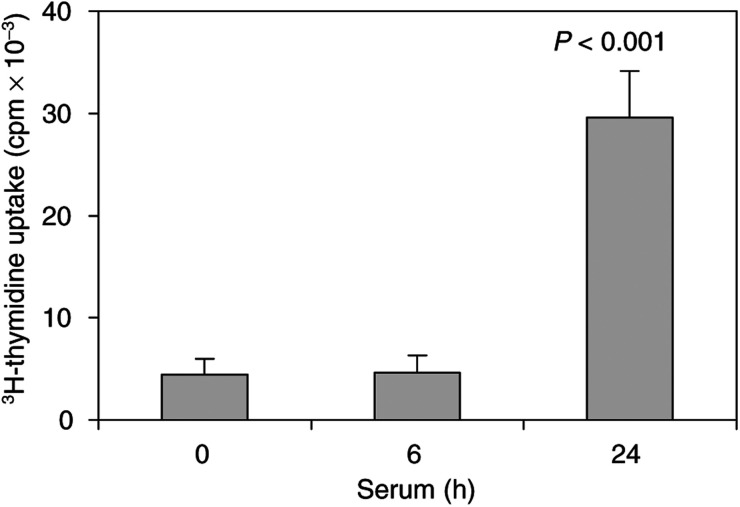
^3^H-thymidine uptake in serum-treated hepatocytes. Cells were grown in 96-well plates in DMEM containing 10% FBS. After 24 h, cells were made quiescent by 48 h serum deprivation and then cells were either untreated (0 time point) or were treated with 15% FBS for 6 or 24 h. Cell proliferation was monitored by adding 0.5 *μ*Ci of ^3^H-thymidine to each well for another 3 h. The radioactivity of collected cellular DNA was determined by scintillation counting. Three independent experiments were performed and the results represent means±s.d. of radioactivity counts expressed in c.p.m.

The HTC-IR cells have been a useful system to study signal transduction and gene expression in hepatocyte line grown in culture (Iwamoto *et al*, 1981; Ostrowski *et al*, 2001). We used ^3^H-thymidine uptake to test their proliferative response to serum treatment. Quiescent HTC-IR cells preincubated with ^3^H-thymidine were treated with 15% serum. At given time points, cells were harvested and ^3^H counts were measured by scintillation counter. Results shown in Figure 1

illustrate that increased ^3^H-thymidine uptake into DNA was not seen at 6 h, but there was a strong increase in uptake 24 h after serum treatment. This ^3^H-thymidine uptake kinetics is similar to studies in other hepatoma cell lines (Kadowaki and Kitagawa, 1988). These results show that the HTC-IR cells are a suitable system to study mitogenic responses to serum treatment.

**Figure 2:**
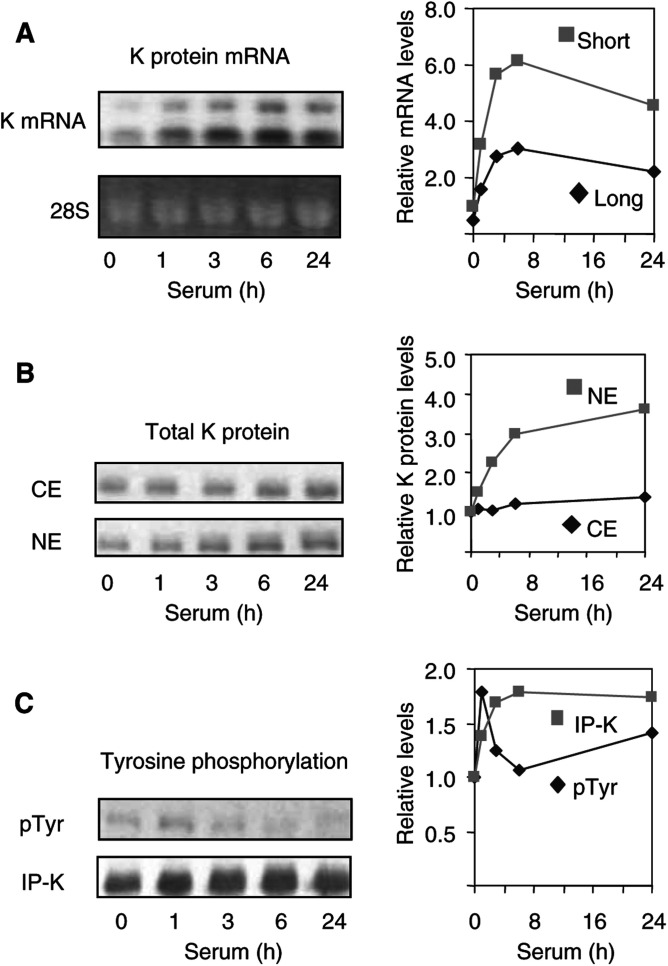
K protein expression in serum-treated hepatocytes. After 48 h of serum deprivation, HTC-IR cells were treated with 15% FCS. At given time points, cells were harvested, and total RNA and proteins as well as nuclear and cytoplasmic proteins were extracted. (**A**) Total RNA was electrophoresed in a 1% agarose/formaldehyde gel and RNA was visualised with ethidium bromide and photographed with digital camera (DC40, Kodak). After RNA transfer, the nylon membranes were probed with ^32^P-labelled K protein cDNA. Autoradiographs were photographed with a digital camera (DC40, Kodak). Densitometric analysis was carried out using OptiQuant™ Image Analysis Software (Packard meriden, CT, USA). The mRNA levels shown in the graph were normalised to the levels of 28S. (**B**) Protein extracts were resolved by SDS–PAGE followed by Western blotting with anti-K protein antibody. Blots were scanned, and densitometric analysis of K protein bands was performed using OptiQuant™ Image Analysis Software. (**C**) Anti-K protein immunoprecipitates from total cell extracts (300 *μ*g protein) were resolved by SDS–PAGE and immnunobloting was performed with either anti-phosphotyrosine (pTyr) or anti-K protein (K) antibodies. Densitometric analysis was done as in (B).

To test if induction of cell cultures to divide alters K protein expression, serum-deprived hepatoma HTC-IR cells were treated with 15% FCS. At given time points, cells were harvested and RNA and protein extracts were prepared (Figure 2

). Northern blot analysis revealed two constitutively expressed K protein transcripts, 2.2 and 3.0 kb. The level of expression of the longer transcript was lower. Serum treatment of resting HTC-IR cells increased the level of both transcripts. The increase was first seen after 1 h of stimulation, peaked at 6 h and decreased after 24 h of serum treatment (Figure 2A). At peak levels (6 h), there was a six-fold increase in both the short and long transcript in response to serum treatment. These results demonstrate that induction of cell proliferation is associated with increased K protein gene expression.

Total cytoplasmic and nuclear extracts (50 *μ*g each) were analysed by SDS–PAGE and anti-K protein immunoblotting. Western blot analysis revealed that serum treatment induced a sustained increase in K protein levels in both cytoplasmic and nuclear fractions (Figure 2B). The increase was far more pronounced in the nuclear fractions where 24 h following treatment there was a 3.6-fold increase in K protein levels compared to resting cells. At the same time point, K protein level in cytoplasmic extracts increased by 40%. These results suggest that the serum-induced increase in K protein mRNA levels (Figure 2A) results in increased K protein synthesis and that most of the newly synthesised K protein is directed to the nucleus.

K protein is tyrosine phosphorylated by Src-family of kinases (Ostrowski *et al*, 2000a; Ostareck-Lederer *et al*, 2002). Serum treatment increases tyrosine phosphorylation of many proteins involved in signal transduction and gene expression (Wang, 1994). To test if K protein is tyrosine phosphorylated in response to a mitogenic signal, K protein was immunoprecipitated with anti-K protein from whole-cell lysates of quiescent and serum-treated HTC-IR cells and the immunoprecipitates were analysed by anti-phosphotyrosine and anti-K protein immunoblots (Figure 2C). There was constitutive tyrosine phosphorylation of K protein in quiescent cells, and following serum treatment the level of tyrosine phosphorylation of K protein increased transiently. Serum treatment increased the total amount of immunoprecipitated K protein, but unlike tyrosine phosphorylation this increase was sustained. The increased level of immunoprecipitated K protein from total cell lysates largely reflects the increase in nuclear K protein (Figure 2B).

### Expression and subcellular distribution of K protein in injured livers

Partial hepatectomy leads to hepatic regeneration resulting in almost complete restoration of the liver mass (Taub, 1996). The mechanisms initiating and controlling hepatocyte ‘priming’ involve activation of several nonspecific factors in the initiating phase. These cellular events include increased Na^+^ flux into the cells, elevation in cAMP content, activation of ornithine decarboxylase and Na^+^/K^+^-ATPase, expression of and response to growth factors and cytokines, activation of transcription factors (NF-*κ*B, Stat-3, AP-1, C/EBP-*β*) and induction of immediate-early genes (e.g. c-*fos*, c-*jun*, c-*myc*) (Taub, 1996; Rozga, 2002; Zimmermann, 2002).

**Figure 3:**
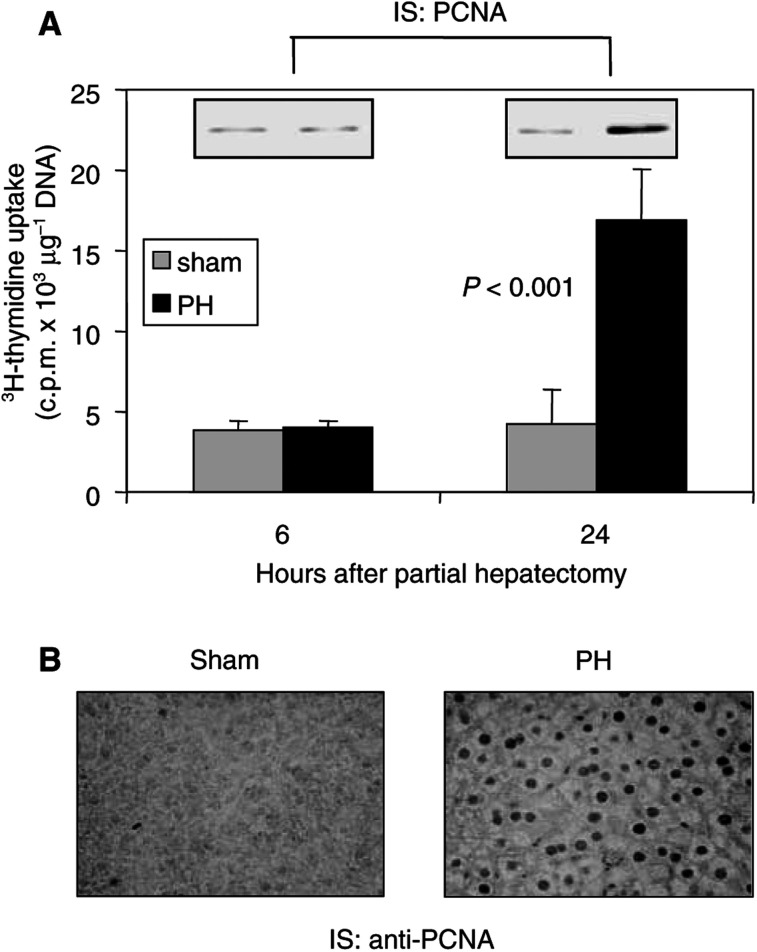
Cell proliferation indices in liver after partial hepatectomy of anaesthetised mice. (**A**) Mice after sham or partial hepatectomy (PH) (three animals in each group at 6 and 24 h after surgery) were given 25 *μ*Ci of ^3^H-thymidine intraperitoneally 1 h prior to euthanasia. The livers were harvested, hepatic DNA was extracted and the radioactivity of 100-*μ*g samples of total hepatic DNA was determined by scintillation counting. Total DNA was measured using a spectrophotometer. The results represent means±s.d. of radioactivity counts expressed in c.p.m. *μ*g^−1^ DNA. The insets represent Western blot analysis of PCNA in liver lysates. (**B**) Immunocytochemistry of the liver tissues was performed as previously described (Kupryjanczyk *et al*, 2003). Briefly, portions of the liver tissues from sham-operated mice and animals following PH (24 h) were embedded in paraffin. After heat-induced epitope retrieval, sections were incubated with anti-PCNA antibody (1 : 4000, Santa Cruz) for 1 h at room temperature. Biotinylated goat anti-mouse IgG was used as a detection system.

^3^H-thymidine uptake into the DNA was used to assess hepatocyte proliferation following partial hepatectomy. Mice were anaesthetised, and after laparatomy in one group of animals the livers were left alone (sham operated), while in the other group the left lobe of the liver was removed (30% hepatectomy)–partial hepatectomy group. Abdominal cavities were closed and the animals were allowed to recover from the ether anaesthesia. At 1 h prior to harvesting the livers, mice were injected with ^3^H-thymidine, animals were again ether anaesthetised and the livers were removed. As shown in Figure 3A

, at 6 h following surgery, no differences in ^3^H-thymidine uptake into the livers were seen between sham and partial hepatectomy animals. In contrast, hepatic DNA synthesis was induced 24 h after partial hepatectomy, but no increase was seen in the control sham-operated mice. Expression of proliferating cell nuclear antigen (PCNA) correlates well with hepatocyte proliferation (Wolf and Michalopoulos, 1992). Proliferating cell nuclear antigen protein levels were determined in liver lysates at 6 and 24 h following surgery by immunoblot analysis. A representative blot is presented as an inset in Figure 3A. Not unexpectedly, the increase in the partial hepatectomy-induced DNA synthesis was mirrored by the increased PCNA expression. Liver lysates contain proteins from hepatocytes and other cell types. Thus, next we did PCNA immunocytochemistry of the livers from either sham-operated animals or animals following partial hepatectomy that allows direct visualisation of hepatocytes. In sham-operated mice there was little or no PCNA detected. In contrast, there was very strong nuclear PCNA staining of hepatocytes 24 h following partial hepatectomy (Figure 3B). These observations are consistent with previous partial hepatectomy studies carried out in rats and mice where DNA synthesis occurred in a single peak 18–24 h after resection (Lambotte *et al*, 1997; Freeman *et al*, 1999).

**Figure 4:**
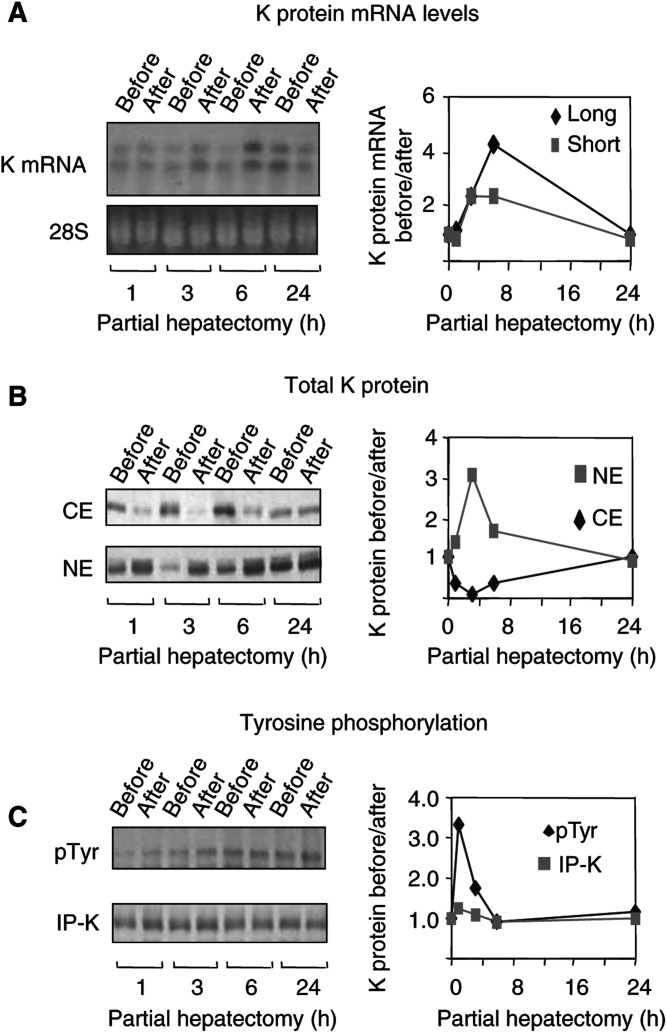
K protein expression after partial hepatectomy in mice. (**A**) Total RNA was extracted from resected lobes (before) and from the remnant livers (after). Northern blots were probed with ^32^P-labelled K protein cDNA. The slower (long) and the faster (short) K protein mRNA transcripts (upper gel) were analysed by densinometry. The intensity of the K protein mRNA bands was normalised to the levels of 28S RNA (lower gel). The results in the graph are expressed as a ratio of levels after (after) to before (before) partial hepatectomy. (**B** and **C**) Protein extracts prepared from livers before and after partial hepatectomy were analysed by anti-K protein Western blots as in Figure 1. Results are expressed as a ratio of levels measured after (after) and before (before) partial hepatectomy of the same liver. (**B**) Levels of K protein in cytoplasmic (CE) and nuclear (NE) extracts. (**C**) Anti-K protein immunoprecipitates from whole-cell lysates analysed with anti-phosphotyrosine (pTyr) and anti-K protein (K) antibody.

As partial hepatectomy is an excellent model of cellular proliferation in an intact organ, we used it to correlate changes in cell proliferation with K protein expression and subcellular distribution in this system (Figure 4

). Mice were anaesthetised, and after laparatomy the left lobe of the liver was removed (30% hepatectomy) and rapidly frozen. Abdominal cavities were closed and the animals were allowed to recover from the ether anaesthesia. At given time points, mice were again ether anaesthetised and the remnant livers were harvested and rapidly frozen. Total RNA, total cell lysates, cytoplasmic and nuclear extracts were prepared from the resected lobes and from the remnant livers. All analyses presented were carried out by comparing levels in the remnant liver to the levels measured in the resected lobe of the same animal (Figure 4).

As in case of HTC-IR cells (Figure 2A), Northern blot analysis showed that in an intact liver there is constitutive expression of the two transcripts of K protein (Figure 4A). The level of both transcripts increased after resection, a change first seen in the remnant liver 3 h after resection; the peak effect occurred at 6 h and the mRNA levels came down to baseline 24 h postpartial hepatectomy. These results show transient increase in K protein mRNA levels that is associated with cell proliferation *in vivo*.

Western blot analysis revealed that there was a transient increase in nuclear K protein, with peak levels (three-fold increase) seen 3 h postpartial hepatectomy (Figure 4B). Concurrently, there was a decrease in cytoplasmic K protein levels. At 24 h postpartial hepatectomy, the levels of K protein in the cytoplasmic and nuclear extracts from remnant livers returned to baseline. These results revealed that following partial hepatectomy, there is nuclear translocation of K protein from the cytoplasm.

Liver injury activates tyrosine kinases leading to phosphorylation of such key factors as the cyclins (Diehl and Rai, 1996; Spiewak Rinaudo and Thorgeirsson, 1997). We used immunoprecipitation assays to assess whether partial hepatectomy alters the level of tyrosine phosphorylation of K protein (Figure 4C). Anti-phosphotyrosine blots of K protein immunoprecipitations showed that in the intact liver there is constitutive tyrosine phosphorylation of K protein. Liver resection induced a transient increase in the level of K protein tyrosine phosphorylation, with the peak effect (3.5-fold increase) observed in the remnant liver 1 h after resection (Figure 4C, pTyr). Since K protein transcripts were transiently induced postpartial hepatectomy (Figure 4A), it was surprising to see that the level of K protein immunoprecipitated from total cell lysates from remnant livers did not change appreciably (Figure 4C, IP-K).

### Expression and subcellular distribution of K protein in hepatic neoplasms

**Figure 5:**
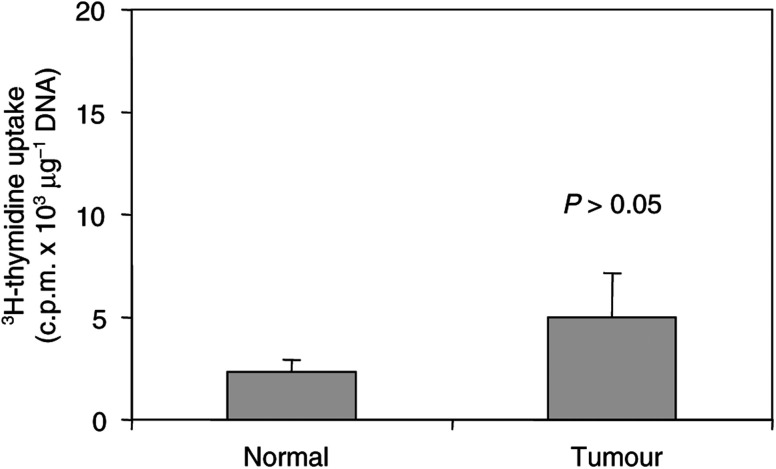
^3^H-thymidine uptake in mouse spontaneous hepatic adenocarcinoma hepatocellular carcinomas. Mice harbouring spontaneous hepatocellular carcinomas (five animals) were given 25 *μ*Ci of ^3^H-thymidine intraperitoneally 1 h prior to euthanasia. The livers were harvested and hepatic DNA was extracted from the liver tumours and from the normal surrounding tissues. Radioactivity of 100-*μ*g samples of total hepatic DNA was determined by scintillation counting. The results represent means±s.d. (*n*=5) of radioactivity counts expressed in c.p.m *μ*g^−1^ DNA.

CBA-T6/W mice develop spontaneous liver tumours including hepatocellular adenoma and carcinoma (Ostrowski *et al*, 2000c). To assess the rates of proliferation of these tumours, we again used ^3^H-thymidine incorporation (Figure 5

). At 1 h after injecting ^3^H-thymidine, mice were ether anaesthetised, the livers were resected and carcinomas were dissected out from the normal liver parenchyma. DNA was purified from normal and cancer tissue and ^3^H counts were assessed as before. A part of the resected tissue was used for histology. Although ^3^H-thymidine incorporation tended to be higher in hepatocellular carcinomas than in the surrounding histologically normal hepatic tissue, these differences were not statistically significant. This is not surprising since these tumours are slow growing (Szymanska, 1991).

**Figure 6:**
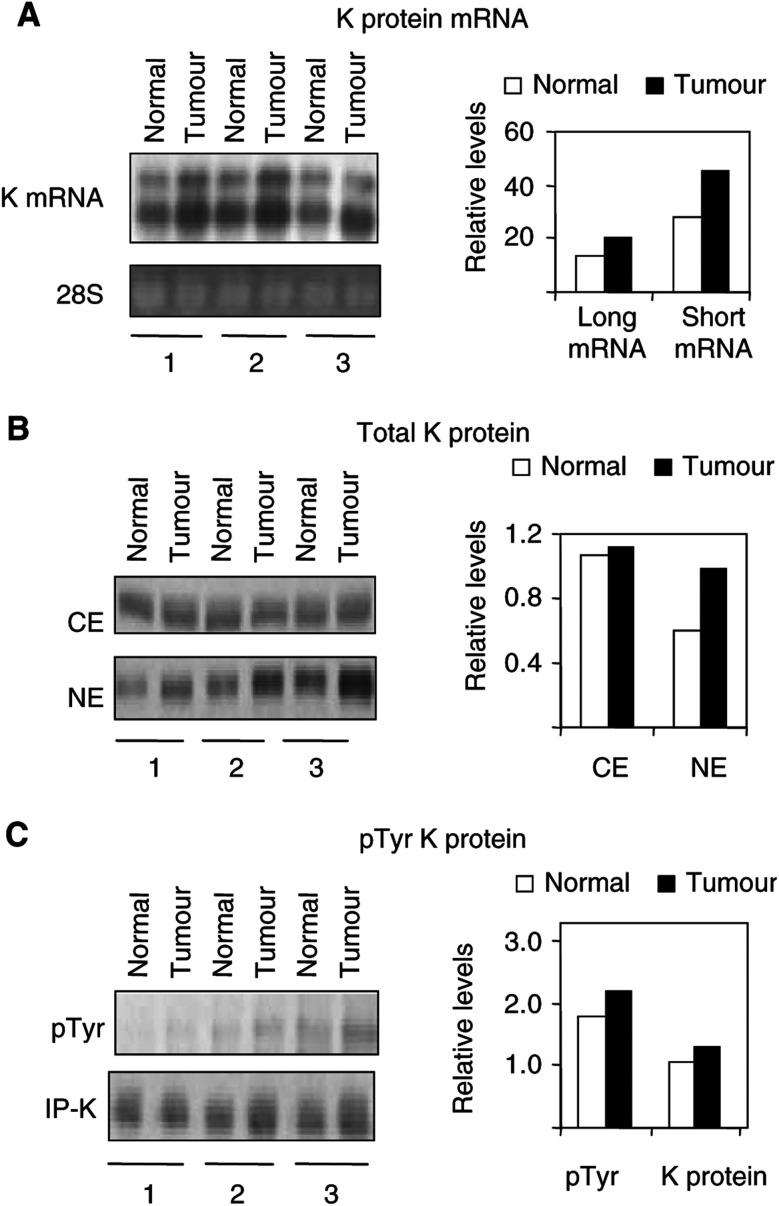
K protein expression in spontaneous hepatic adenocarcinoma in mice. (**A**) Total RNA was extracted from hepatic adenocarcinoma and the livers harbouring these tumours. Northern blots were probed with ^32^P-labelled K protein cDNA. The slower (long) and the faster (short) K protein mRNA transcripts (upper gel) were analysed by densinometry. The intensity of the K protein mRNA bands was normalised to the levels of 28S RNA (lower gel). The results in the graph are expressed as a ratio of levels measured in the adenocarcinoma (tumour) and those measured in the liver (normal) harbouring the tumour. (**B** and **C**) Protein extracts prepared from liver adenocarcinomas (tumour) and normal liver tissue (normal) surrounding these tumours were analysed by anti-K protein Western blots as in Figure 1. Results are expressed as the ratio of levels measured in the tumour (tumour) and those in the normal parenchyma (normal) of the same liver. (**B**) Levels of K protein in cytoplasmic (CE) and nuclear (NE) extracts. (**C**) Anti-K protein immunoprecipitates from whole-tissue extracts analysed with anti-phosphotyrosine (pTyr) and anti-K protein (K) antibody.

We used the mice with hepatocellular carcinoma to assess K protein expression and its subcellular distribution. The Livers harbouring tumours were resected from anaesthetised mice and carcinomas were dissected out from the normal liver parenchyma. As before, a part of the resected tissue was used for histology and the rest was rapidly frozen. Figure 6

shows finding in three animals with hepatocellular carcinoma. Northern blot analysis revealed two transcripts, and in all three animals the levels of K protein mRNA were higher in the tumour than in the surrounding normal parenchyma (Figure 6A). The increased K protein mRNA levels in cancer is consistent with the findings in serum-treated HTC-IR (Figure 2A) and in livers postpartial hepatectomy (Figure 4A).

In further agreement with the other results (Figures 2 and 4), Western blots showed that in all the three animals the level of K protein in nuclear extracts was higher in the tumour than in the normal liver harbouring these cancers (an average 66% higher in the tumour) (Figure 4B). In contrast, the levels of K protein in cytoplasmic extracts from cancer were not different from the normal parenchyma. In all the three animals, the level of tyrosine phosphorylation of K protein in the tumours was higher than in the normal liver (average 20% difference), but in part this might be explained by the slightly higher levels of K protein immunoprecipitated from the cancer (Figure 6C).

**Figure 7:**
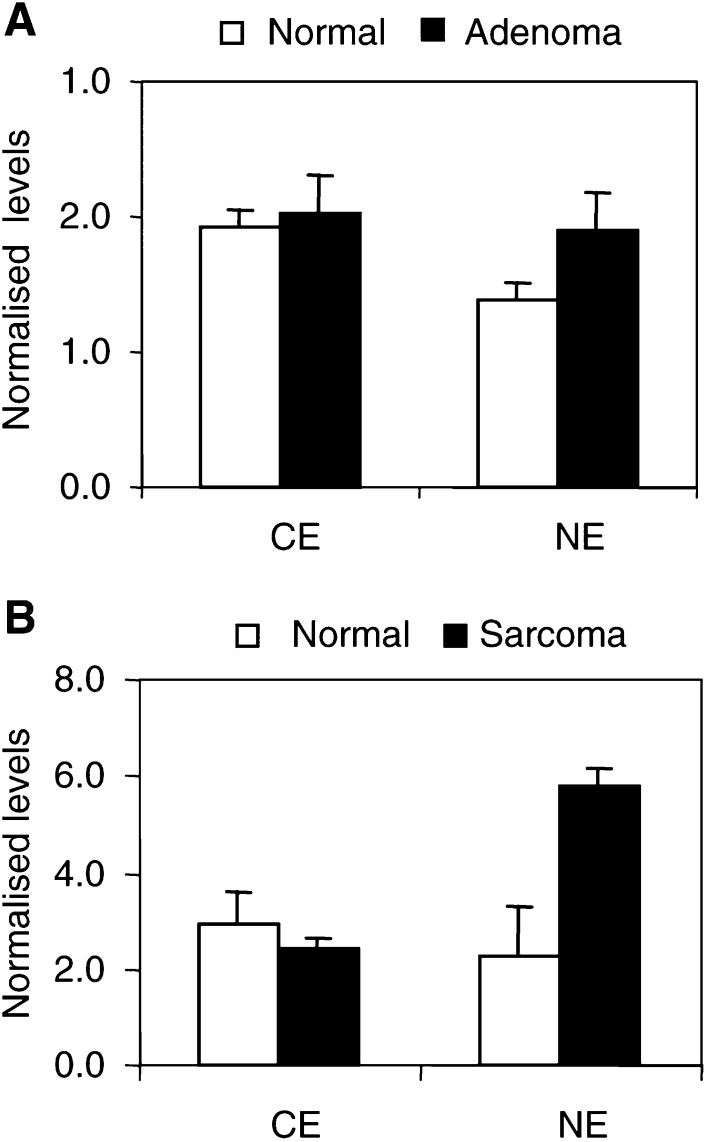
Expression of K protein in spontaneous adenomas and implanted sarcomas in mouse livers. Cytoplasmic (CE) and nuclear (NE) extracts were prepared from spontaneous liver adenomas (adenoma), implanted sarcomas (sarcoma) and normal liver parenchyma (normal) surrounding these tumours. Levels of K protein were analysed in Western blots as in Figure 1. Results are expressed as the ratio of levels measured in the tumour (tumour) and those in the normal tissue (normal) of the same liver.

We also assessed K protein expression and subcellular distribution in hepatic adenoma (seven animals) and in L1 sarcoma implanted into liver (six animals) (Figure 7

). The level of K protein in the cytoplasmic extracts from liver adenomas was the same as in the adjacent normal parenchyma (Figure 7A, CE). In six out of seven animals, the level of nuclear K protein was higher in the hepatic adenomas compared to the normal liver parenchyma surrounding the tumours (Figure 7A, NE). On an average, the level of K protein in the adenomas was 38% higher than in normal tissues. In the implanted sarcomas, which are more aggressive tumours, in all the six animals the level of nuclear K protein was higher than in the nuclear extracts from livers harbouring these tumours (Figure 7B). On an average, K protein levels in the nuclear extracts from sarcomas was 2.5-fold higher than those measured in nuclear extracts from normal liver parenchyma adjacent to these tumours. As in the hepatocellular neoplasms (Figures 6 and 7A), the levels of K protein in cytoplasmic extracts from sarcomas and normal liver tissues were not different (Figure 7B).

### Activation of c-*myc* gene expression in serum-treated HTC-IR cells, in injured livers and liver tumours

Mitogenic stimulation leads to rapid induction of immediate-early genes (Herschman, 1991). Immediate-early genes are activated in a protein synthesis-independent manner and are involved in cell proliferation (Jochum *et al*, 2001). They regulate later phases in *G*_1_ of the cell cycle and represent diverse classes of genes including those encoding transcription factors, c-*myc* is one of the immediate-early genes, whose expression is transiently increased in G_1_. c-*myc* encodes a transcription factor that targets a host of genes that regulate cell proliferation (Bouchard *et al*, 1998). With regard to the liver, antisense oligomer to c-*myc* can reduce cell proliferation in the regenerating rat liver, suggesting that expression of this gene plays a role in the process of regeneration (Arora *et al*, 2000). In response to serum treatment, K protein is recruited to multiple sites along the c-*myc* locus (Ostrowski *et al*, 2003a). In gene reporter systems, K protein was shown to regulate c-*myc* promoter (Michelotti *et al*, 1995,^1996^) and translational elements activity (Evans *et al*, 2003). Studies in breast carcinoma lines suggested that increased expression of K protein enhanced c-Myc levels (Mandal *et al*, 2001). Taken together, these studies suggest that K protein regulates c-*myc* gene expression on both transcriptional and post-transcriptional levels.

**Figure 8:**
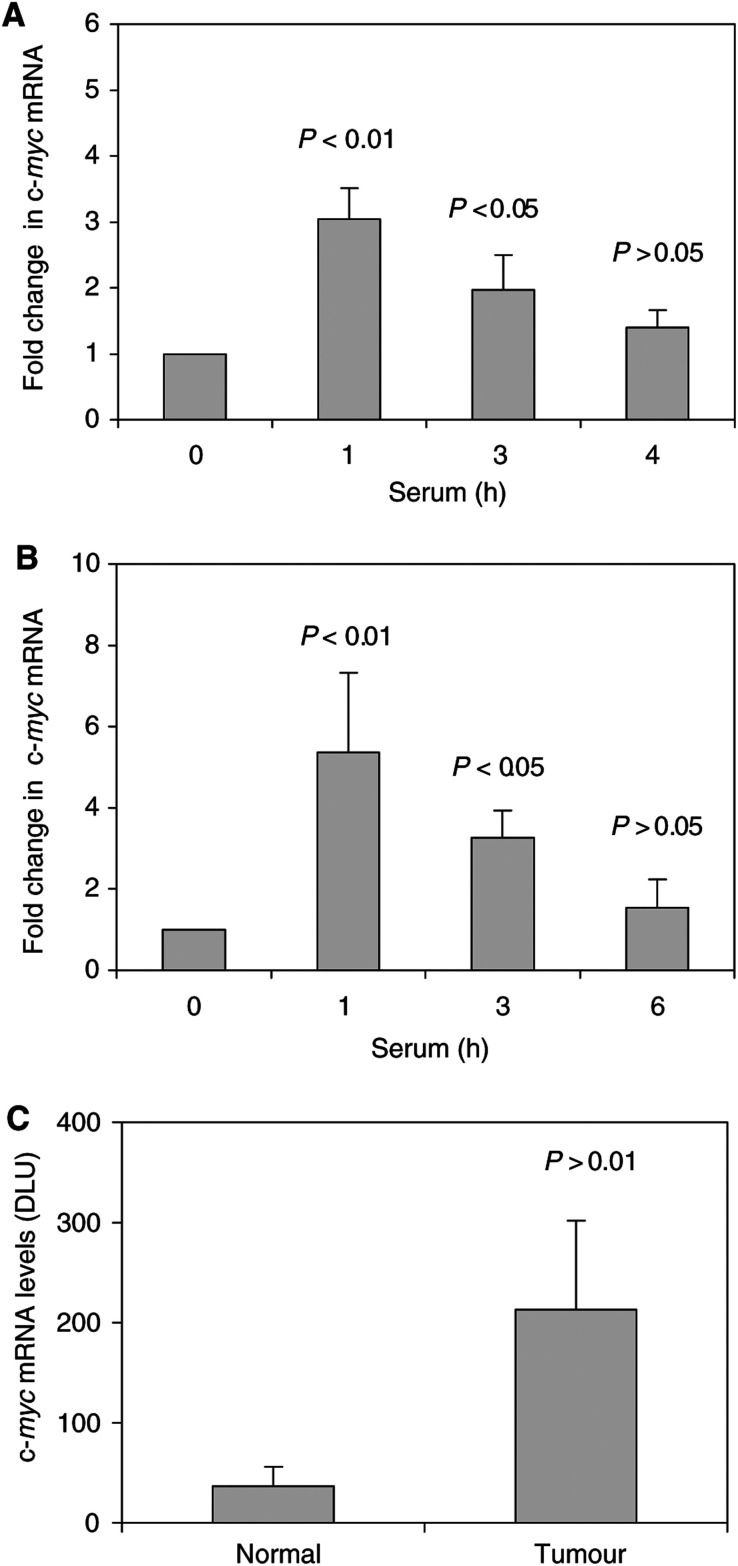
c-*myc* mRNA transcript levels in serum-treated hepatocyte cultures (**A**), postpartial hepatectomy (**B**) and in hepatic tumours (**C**). (**A**) Serum-deprived HTC-IR cells were treated with 15% FBS for 0, 1, 3, 6 and 24 h. After harvesting the cells, total RNA was isolated and used in RT. PCR was carried out using [*α*^32^P]dCTP (0.5 *μ*Ci per reaction) and c-*myc* primers (forward: GCAAATGCTCCAGCCCCAGGTC; reverse: AGTCCCAAAGCCCCAGCCAAGGTT). PCR products were resolved by native PAGE and were quantified using a phosphorimager (Cyclone, Packard meriden, CT, USA). Results are expressed as fold increase in the mRNA level at each time point of serum treatment compared to the levels seen in untreated cells. The values represent means±s.d. (*n*=3). (**B**) Partial hepatectomy and control sham operation were carried out as in Figure 3. At given time points following surgery, the livers were harvested from each group and total RNA was isolated (three animals for each time point), c-*myc* mRNA levels were determined by RT–PCR as above. Results are expressed as fold increase in mRNA levels compared to sham-operated mice. (**C**) Comparison of c-*myc* mRNA levels in spontaneous hepatocellular carcinoma and the normal surrounding liver tissue, c-*myc* mRNA levels were determined by RT–PCR and the ^32^P signal of the labelled products is expressed in DLU. The results represent means±s.d. (*n*=5 animals).

We next compared semiquantitative RT–PCR analysis of RNA in serum-treated HTC-IR cells, in resected livers and in hepatic tumours to correlate c-*myc* transcript levels with K protein expression in these models (Figure 8

). Treatment of serum-starved HTC-IR cells rapidly induced the c*-myc* expression (Figure 8A), with a peak in c-*myc* expression seen at 1 h after serum treatment. This time course is similar to the kinetics of serum-induced transient recruitment of K protein to the promoter and transcribed region of the c-*myc* locus in these cells (Ostrowski *et al*, 2003a). The kinetics of c-*myc* mRNA expression after partial hepatectomy (Figure 8B) was similar to that seen in cultured serum-treated hepatocytes *in vitro*; a large peak in c-*myc* transcript levels was seen at 1 h following partial resection, and its level declined nearly to baseline at 6 h. These c-*myc* results are similar to the studies published by others (Taub *et al*, 1987). Similarly, there was also increased c-*myc* transcript expression in hepatocellular carcinoma compared to normal tissue, that is, an eight-fold higher levels (Figure 8C).

## DISCUSSION

Treatment of resting HTC-IR cells with serum resulted in sustained increase in nuclear K protein with little changes in cytoplasmic K protein (Figure 2B). Similar observations were made in hepatic tumours (Figures 6B and 7). While the levels of K protein in nuclear extracts were higher in the neoplasms compared to the adjacent normal parenchyma, the levels of K protein in the cytoplasmic fractions were the same in the neoplastic and the normal surrounding tissues. Serum-treated HTC-IR cells and tumours represent sustained states of enhanced cell proliferation. In contrast, following partial hepatectomy, the number of cycles of cell division is limited. Within minutes after partial hepatectomy, quiescent liver cells in the remaining lobes enter a state of replicative competence (an initiation phase), followed by a proliferation phase and, finally, by a termination phase (Zimmermann, 2002). Thus, it is not surprising that postpartial hepatectomy there was only a transient nuclear shift of K protein that peaked at 3 h following liver injury and by 6 h it was near baseline. The kinetics of the nuclear shift of K protein in response to serum treatment and following liver injury provides some clues regarding the consequences of this effect. In response to serum treatment, K protein is recruited to multiple sites along the c-*myc* locus including both the promoter and the transcribed regions (Ostrowski *et al*, 2003a). K protein binds *in vitro* CT-rich DNA from the c-*myc* promoter (Michelotti *et al*, 1995) and activates c-*myc* promoter activity in several types of cells including breast cancer (Michelotti *et al*, 1996; Mandal *et al*, 2001). Here, we have shown that c-*myc* expression is activated in hepatocyte culture in response to mitogens and following liver injury (Figure 8A and B). Taken together, these results suggest that at an early time point following liver injury K protein may play a role in the induction of c-*myc* gene. Expression of c-*fos* and *egr-1* is also activated in remnant liver following partial hepatectomy (Taub, 1996). Promoters of both c-*fos* and *egr-1* genes contain putative CT-like K protein-binding motifs, and in response to serum treatment of hepatocyte culture K protein is recruited to both *egr-1* (Ostrowski *et al*, 2003a) and c-*fos* (unpublished observations) loci. It is, therefore, plausible that the role of the K protein fraction that is newly directed to the nucleus in remnant livers is to regulate transcription of these immediate-early genes and other factors that mediate the enhanced cell proliferation during an initiation (prereplicative) phase. Here, the increase in the level of tyrosine phosphorylation of K protein (Figure 4C) may play a role in its nuclear translocation and/or its recruitment to inducible transcribed gene loci such as the immediate-early genes (Ostrowski *et al*, 2003a).

Increased K protein expression has previously been found in breast cancer cells. In that study, the authors provide evidence that the increased K protein levels contribute to the enhanced c-*myc* gene expression in these tumours (Mandal *et al*, 2001). The current study demonstrates for the first time that there is a nuclear shift of K protein in hepatic neoplasm and other states of enhanced proliferation. As discussed above, there is considerable evidence that K protein regulates c-*myc* gene expression, so the increased level of K protein in the nucleus seen in tumours (Figures 6 and 7) may play a role in enhanced expression of this immediate-early gene in malignancy. K protein binding appears to be genomewide, where it exhibits both constitutive and inducible interactions with chromatin (Ostrowski *et al*, 2003a). Some of these interactions may reflect direct binding of K protein to DNA. However, K protein binds many proteins, DNA and RNA (Bomsztyk *et al*, 1997). Some of the K protein partners are recruited to transcribed loci by interacting with DNA, transcriptionally active complexes or with nascent RNA. One or more of these K protein interactions could be responsible for the recruitment of K protein to chromatin by an indirect mechanism. Thus, the nuclear shift of K protein in tumours (Figures 6 and 7) may reflect its involvement in multiple DNA- and/or RNA-directed processes that are altered in malignancies. With respect to nuclear K shift in cancer, two nuclear processes seem relevant to consider. First, cisplatin cross-linking in human breast cancer cells revealed that K protein is one of the nuclear matrix proteins (Samuel *et al*, 1998). The key role of nuclear matrix is to organise the chromatin topology so that it can serve as a conducive template for transcription and replication. Although much remains to be learned, abnormal nuclear matrix is one of the morphologic hallmarks of cancer (Leman and Getzenberg, 2002). Thus, the nuclear shift of K may reflect, in part, altered protein composition of nuclear matrix in cancer. If so, the ability of cisplatin to cross-link K protein to DNA may be one of the mechanisms by which this chemotherapeutic agent inhibits processes that compose transcription and replication (Samuel *et al*, 1998). Second, K and other hnRNP proteins bind mammalian telomeric sequences (Lacroix *et al*, 2000; Ford *et al*, 2002) and *Saccharomyces cerevisiae* K protein-like genes regulate telomeric processes (Denisenko and Bomsztyk, 2002). These observations suggest that K protein may be involved in maintaining the integrity of mammalian telomeres. Preservation of telomere length which, in part, results from reactivation of telomerase is another hallmark of continuous cells growth, especially of advanced malignancies (Neumann and Reddel, 2002). The increased nuclear levels of K protein may play a role in the altered telomeric processes seen in cancer.

In summary, we have demonstrated that in several states of enhanced cell proliferation, there are increased K protein levels in the nucleus. Induction of cell proliferation results in the activation of a large repertoire of genes (Iyer *et al*, 1999). K protein is an abundant factor involved in transcription, mRNA processing and other events that compose gene expression. It is likely that the increased K protein levels seen in the nuclei of the proliferating cells serve to support nuclear process that not only composes inducible expression of a very large number genes but also maintains conducive chromatin topology in growing cells.
